# Naturally Occurring Compounds: New Potential Weapons against Oxidative Stress in Chronic Kidney Disease

**DOI:** 10.3390/ijms18071481

**Published:** 2017-07-10

**Authors:** Lorenzo Signorini, Simona Granata, Antonio Lupo, Gianluigi Zaza

**Affiliations:** Renal Unit, Department of Medicine, University-Hospital of Verona, Verona 37126, Italy; docsignorini88@gmail.com (L.S.); simona.granata@univr.it (S.G.); antonio.lupo@univr.it (A.L.)

**Keywords:** oxidative stress, reactive oxygen species, chronic kidney disease, antioxidants, nephrology

## Abstract

Oxidative stress is a well-described imbalance between the production of reactive oxygen species (ROS) and the antioxidant defense system of cells and tissues. The overproduction of free radicals damages all components of the cell (proteins, lipids, nucleic acids) and modifies their physiological functions. As widely described, this condition is a biochemical hallmark of chronic kidney disease (CKD) and may dramatically influence the progression of renal impairment and the onset/development of major systemic comorbidities including cardiovascular diseases. This state is exacerbated by exposure of the body to uremic toxins and dialysis, a treatment that, although necessary to ensure patients’ survival, exposes cells to non-physiological contact with extracorporeal circuits and membranes with consequent mitochondrial and anti-redox cellular system alterations. Therefore, it is undeniable that counteracting oxidative stress machinery is a major pharmacological target in medicine/nephrology. As a consequence, in recent years several new naturally occurring compounds, administered alone or integrated with classical therapies and an appropriate lifestyle, have been proposed as therapeutic tools for CKD patients. In this paper, we reviewed the recent literature regarding the “pioneering” in vivo testing of these agents and their inclusion in small clinical trials performed in patients affected by CKD.

## 1. Introduction

Oxidative stress, a biochemical imbalance between reactive oxygen species (ROS) production and antioxidant defenses, has been reported as an important biochemical hallmark of several human diseases.

In chronic kidney disease (CKD), this deregulated biochemical machinery has been associated with disease progression and with the onset/development of severe systemic complications (mainly atherosclerosis and other cardiovascular diseases), with a consequent remarkable impact on major clinical outcomes [1,2,3,4,5,6].

Although present from the early stages of renal impairment, it appears severely enhanced with advancing stages of CKD and the start of renal replacement therapy (hemodialysis or peritoneal dialysis) [3].

Several biological factors are responsible for oxidative stress in this large population of patients, but, as recently described, mitochondrial deregulation seems to have a primary role [7,8,9].

Mitochondria are organelles with a major role in generating energy for cellular metabolism by the oxidative phosphorylation system (OXPHOS), and they are involved in several physiological cellular functions (e.g., ion homeostasis, heme and steroid synthesis, calcium signaling, apoptosis [10,11,12,13]). During CKD mitochondria may undergo a profound deregulation that can induce functional alterations. CKD is associated with a decline in mitochondrial content at the early stage of the disease [14].

In particular, during renal damage, continuous mitochondrial insults may activate a vicious functional circle responsible for an overproduction of ROS which can induce mitochondrial DNA mutations, damage the mitochondrial respiratory chain, alter membrane permeability, and influence Ca^2+^ homeostasis and mitochondrial defense systems. Mitochondrial dysfunction and renal failure are strictly connected: it has been demonstrated that the role of mitochondria in podocyte injury leads to proteinuria [15,16] as well as epithelial to mesenchymal transition of tubular epithelial cells [17,18]. Moreover, uremic toxins impair OXPHOS in epithelial tubular cells [19].

Other biological factors contributing to oxidative stress in CKD are the enzymatic complex NADPH oxidase and the xanthine oxidase pathway. NADPH oxidase catalyzes the transfer of electrons to oxygen to produce superoxide anion (O_2_^−^) that is immediately converted to H_2_O_2_. These, in turn, are precursors of other ROS that cause damages to proteins, lipids, and nucleic acids [20]. NADPH oxidase-derived ROS are involved in cell signaling, ion channel activity, gene expression, and in the direct killing of invading microbes in phagocytes. Its activity is upregulated in all stages of CKD and in dialysis [21,22,23].

Xanthine oxidase catalyzes the oxidation of hypoxanthine to xanthine and xanthine to uric acid together with ROS release. Xanthine oxidase activity is higher in CKD patients [24] and could be an independent predictor of cardiovascular events in CKD and hemodialysis patients [25]. In this context, allopurinol, the xanthine oxidase inhibitor acting as a competitive substrate for the enzyme, decreases serum uric acid levels and its toxic effects [26]. Several studies have shown that allopurinol treatment decreases C-reactive protein (CRP) levels, slows the progression of renal disease, decreases the number of hospitalizations, and reduces cardiovascular risk [27,28,29,30]. However, since it is excreted by urine, it requires individual dosage modification in CKD patients, and consequently may have poor control over the serum concentration of uric acid [31,32]. In addition, systematic reviews have highlighted that allopurinol could have only a partial therapeutic efficacy, and may also induce adverse effects in CKD patients [33,34].

Febuxostat, an orally administered nonpurine selective inhibitor of xanthine oxidase with two excretion pathways (urinary and fecal), was more effective in the reduction and maintenance of serum urate levels <6.0 mg/dL compared to allopurinol in patients with renal impairment [35,36,37].

At the same time, antioxidant systems are defective in CKD [38,39,40,41], but we recently described that nuclear factor erythroid 2-related factor 2 (NRF-2) and one of its target genes, superoxide dismutase (SOD) 2, are upregulated in dialysis-treated patients, defining a possible antioxidant system able to contrast ROS production.

Therefore, a worldwide interest is emerging in identifying and testing compounds able to significantly counteract oxidative damage in CKD. In particular, natural compounds targeting mitochondria, alone or combined with conventional therapies and lifestyle modifications, could represent valuable tools to prevent this condition and, because of the low adverse effects, they could be employed in patients undergoing both conservative and dialysis treatment [42].

These compounds are frequently included in the diet at biologically active concentrations and represent essential elements of traditional medicine from several countries. Although effective and relatively safety, they have been only partially investigated in nephrology and their efficacy in CKD is still debated.

To this purpose, a large number of studies have begun to address this objective by employing animal models and small clinical trials.

## 2. l-Carnitine

l-Carnitine (4-*N*-trimethylammonium-3-hydroxybutyric acid) is an amino acid-derived compound obtained mostly through the diet, particularly from animal-derived foods. It is endogenously synthesized in the kidney and liver from lysine and methionine, with a daily rate of 1.2 µmol/kg/day [43].

l-Carnitine acts as a transporter of long-chain fatty acids chains across the mitochondrial inner membrane, through a series of reversible transesterification reactions catalyzed by the group of carnitine acyltransferases. In these reactions, coenzyme A (CoA), bound to fatty acids, is substituted by the hydroxyl group of carnitine, forming acyl-carnitine. This molecule is transported from the cytosol to the inner mitochondrial matrix thanks to the combined activity of carnitine-acyl-carnitine translocase and carnitine palmitoyltransferase I and II. Here, acyl-carnitine is converted again into acyl-CoA, which can enter the β-oxidation and finally generate energy in the electron transport chain [44,45].

Carnitine is thus fundamental for mitochondrial and cellular physiological activity. It has been demonstrated that its deficiency leads to (1) the accumulation of free fatty acids into cells; (2) impaired export of the excess of organic acids, particularly in some of the secondary carnitine insufficiencies, including hemodialysis; and (3) increased mitochondrial dysfunction and ROS production, secondary to acetyl-CoA accumulation and multiple enzymatic inhibition [44,46].

Furthermore, carnitine acts as both a direct and indirect antioxidant by scavenging ROS, chelating iron [47], increasing the expression and activity of antioxidant enzymes [48], and by inhibiting lipid peroxidation and xanthine oxidase activity [49,50].

CKD is characterized by a reduced synthesis of l-carnitine, proportional to the decline of the glomerular filtration rate (GFR). Nevertheless, its level is higher in CKD patients under conservative therapy than in healthy individuals, probably through a compensatory effect of the liver [51].

On the other hand, CKD patients undergoing hemodialysis (HD) and peritoneal dialysis (PD) show a reduction of free l-carnitine levels in plasma and muscle. This is due to several factors, including loss during dialysis treatment, intestinal malabsorption, and the impaired synthetic capacity of the kidney. In particular, Di Liberato et al. suggested a potential role of PD modality (CAPD) on carnitine depletion and the possible advantage of carnitine-containing dialysis fluids [52].

Due to its antioxidant role, the oral administration of l-carnitine to HD patients resulted in an increased glutathione (GSH) level, increased glutathione peroxidase activity, and a decreased malondyaldeide (MDA) level [53,54]. Interestingly, several reports have suggested that its supplementation might have a positive effect on the response to erythropoietin (EPO) in long-term hemodialysis patients [55,56,57,58], but it did not modify EPO requirements in patients new to hemodialysis [59].

During the past few years, some federal agencies have suggested the use of l-carnitine in HD. In 2003, the National Kidney Foundation developed a practice recommendation for the use of l-carnitine in patients with dialysis-related carnitine disorders, most notably erythropoietin-resistant anemia, intradialytic hypotension, cardiomyopathy, and fatigability [60]. However, KDOQI guidelines state that there is not sufficient evidence to recommend the administration of L-carnitine to HD patients with anemia [61].

Two important meta-analyses of the l-carnitine administration in HD patients were recently published, with controversial results. Chen et al. concluded that l-carnitine administration decreases serum low density lipoprotein (LDL) and CRP, while it fails to ameliorate EPO responsiveness and anemia [62]. In contrast Yang et al. did not find any conclusive evidence of effectiveness against inflammation, oxidative stress, anemia, nutrition, dyslipidemia, hyperparathyroidism, or quality of life in HD patients undergoing l-carnitine administration. These contrasting results suggest that additional long-term controlled randomized clinical trials are necessary to definitively understand the clinical utility of carnitine administration in CKD patients [63].

## 3. Vitamin E

Vitamin E encompasses a group of eight chemically related molecules, comprising α, β, γ, δ tocopherol, and the derived tocotrienols [64]. The most biologically active form is α-tocopherol [65]. The main sources of vitamin E are seed oils (wheat germ oil: 150 mg/mL, almond oil: 95 mg/mL, olive oil: 15 mg/mL).

α-Tocopherol maintains the integrity of long-chain polyunsaturated fatty acids in the membranes of cells and thus preserves their bioactivity [66,67]. In particular, it protects lipid structures from peroxidation and increases LDL resistance to oxidative modification [68,69], suggesting a potential effect of vitamin E in the prevention of atherosclerosis [70,71].

Vitamin E is a powerful peroxyl radical scavenger that returns to its reduced state by reacting with vitamin C (or other hydrogen donors) [72].

Moreover, it reduces the mitochondrial generation of hydrogen peroxide and it regulates the expression of genes implicated in inflammation and fibrosis [73,74,75,76].

Although vitamin E therapy has been extensively studied in CKD patients, there is no consensus about the benefit obtained from its administration.

The SPACE study evaluated the cardiovascular protective effects of orally administered, high doses of vitamin E (800 IU/die) on HD patients with previous cardiovascular events, over a period of 519 days. The treatment resulted in a 40% decline in both composite cardiovascular events and myocardial infarction [77].

On the other hand, the administration of vitamin E (400 IU/day) to patients with mild to moderate renal failure failed to obtain beneficial effects on cardiovascular outcomes in the Heart Outcomes Prevention Evaluation (HOPE) study [78].

These contrasting results may be partially due to differences in the enrollment criteria (e.g., the SPACE trial included patients with higher cardiovascular risk, and participants were treated with a higher dose of vitamin E); moreover, most participants in the SPACE trial (43.3% of the vitamin E group) also consumed vitamin C. Finally, the SPACE study enrolled a smaller sample (196 HD patients) compared with other trials, which resulted in large confidence intervals based on a broad composite end-point.

More recent studies have shown the positive effect of vitamin E administration before coronary procedures in the prevention of contrast-induced acute kidney injury in patients with CKD undergoing elective coronary procedures [79,80].

Additionally, long-term use of vitamin E-coated HD filters improved oxidative stress, inflammatory markers, and hemoglobin levels, and reduced Erythropoiesis-Stimulating Agents (ESA) requirement without affecting dialysis adequacy [81,82,83,84,85,86,87].

Vitamin E combined with Pravastatin and homocysteine-lowering therapy was evaluated against vascular stiffness progression over 18 months in patients with mild to moderate CKD. This treatment resulted in significant improvement of vascular compliance and distensibility, decrement in common carotid intima-media thickness, and increase in brachial artery flow-mediated dilatation; however, the effect of other confounding variables was unclear [88,89].

It is noteworthy that vitamin E could have also a pro-oxidant action under special conditions that can be encountered in HD patients [90,91]. In fact, oral α-tocopherol administration (500 mg/day) for 1 year to HD patients caused reduced SOD activity and total antioxidant status [91]. This could be due to the low level of other antioxidants necessary to restore the reduced form of vitamin E (e.g., vitamin C) [92].

Based on research studies, vitamin E supplementation could be a potential valuable adjuvant therapy to contrast oxidative stress and lower lipid peroxidation in CKD and HD patients. Still, because of its adverse effects [93], its clinical employment in nephrology needs to be better defined.

## 4. Vitamin C

Vitamin C (ascorbic acid) is a water-soluble essential antioxidant obtained from citrus fruits and some green vegetables such as broccoli and spinach [94]. Its recommended daily dose is approximately 90 mg in adult men and 75 mg in adult women [95].

Vitamin C prevents oxidative damage by scavenging ROS and reactive nitrogen species [96]. Moreover, it shows an anti-apoptotic activity by maintaining the mitochondrial membrane potential and protecting mitochondrial DNA from oxidant insults [97,98,99,100].

It has been demonstrated that a reduction in both the total vitamin C concentration and the active form (ascorbate) is probably caused by the limited intake of potassium-rich foods in CKD patients in conservative treatment, and by the loss during HD treatment. Another possible explanation is an impairment of enzymatic or non-enzymatic recycling of ascorbate from dehydroascorbate (the oxidized form of vitamin C), since the recycling is largely GSH-dependent [101] and dialysis patients have a marked GSH deficiency [102,103]. The low vitamin C plasma levels in CKD patients has been, then, associated with an increased risk for fatal and nonfatal major adverse cardiovascular events [104,105].

To avoid this condition, currently oral ascorbate (1–1.5 g/week) or parenteral ascorbate (300 mg/dialysis session) are suggested to balance subclinical deficiency [103].

Poly-vitamin supplementation, including vitamin C (250 mg/day), for eight weeks to HD patients did not induce any change in inflammation, malnutrition, or oxidative stress markers [106].

Moreover, in a double-blind randomized clinical trial on CKD patients (stage IV–V and HD), vitamin C (250 mg, three times a week) failed to demonstrate a real clinical benefit when evaluated against uremic symptoms and cardiovascular stability [107].

Conversely, several studies showed advantages of vitamin C administration. In a cross-over study, patients treated for three months with vitamin C (200 mg/day) showed decreased CRP levels and increased serum prealbumin concentration as marker of malnutrition [108]. Interestingly, the use of vitamin E-coated dialysis membranes significantly reduced oxidative stress, avoided a reduction of erythrocyte reductases activity, and decreased the level of proinflammatory cytokines [81]. Moreover, other studies reported that this vitamin improved the response to ESA with a significant enhancement of hemoglobin levels and transferrin saturation [109,110]. At the moment, despite encouraging results from clinical research studies, the use of vitamin C is still debated in nephrology.

## 5. Coenzyme Q10

Coenzyme Q10 (CoQ10) is a high lipophilic molecule diffuse in eukaryotic cells that is mostly concentrated in mitochondria, as component of the mitochondrial respiratory chain where it transports electrons from complex I/II to complex III [111].

It is synthesized in the human body and is assumed with food (animal muscle and liver, blue fish, soy beans, and olive oil) with an estimated daily intake of 3–6 mg [112].

Due to its abundant distribution and intra-membranous localization, CoQ10 prevents membrane lipid peroxidation since hydroxyl and superoxide radicals generated in the membrane during electron transport chain would otherwise rapidly react with neighboring lipid and protein molecules [113,114].

CoQ10 regenerates vitamin E from the α-tocopheroxyl radical and prevents the oxidation of nucleic acids, particularly of mitochondrial DNA [113]. Additionally, it inhibits mitochondria-dependent apoptosis by preventing permeability transition pore opening and mitochondrial membrane potential depolarization [111,115]. Moreover, increased mitochondrial CoQ10 content results in a general improvement of bioenergetic parameters, such as oxygen consumption, ATP content, mitochondrial potential, and protein synthesis [116].

As a consequence, CoQ10 has been studied in clinical settings in order to prevent atherosclerosis, aging, and the progression of chronic diseases such as CKD.

In CKD patients, it an inverse relationship between CoQ10 and renal function has been reported [117,118].

An interesting correlation was also demonstrated between reduced CoQ10 levels and epicardial fat tissue (EFT) in HD patients [119]. EFT is the visceral adipose tissue surrounding the subepicardial coronary vessels. It is an active source of proinflammatory factors [120,121,122], and, in greater amounts, has recently been related to a higher risk for atherosclerosis and heart disease in PD and HD patients [123,124]. Thus, increased EFT in HD patients could cause the consumption of CoQ10 as an antioxidant molecule, and its supplementation was hypothesized able to correct this imbalance, reducing cardiovascular risk.

In a recent trial involving patients in maintenance HD, oral CoQ10 (200 mg/day) supplementation did not induce changes in exercise performance and in the blood level of oxidative system markers compared with a placebo [125].

As with other similar compounds, at present, there is no a clear evidence that justifies the “day by day” clinical employment of CoQ10 in CKD patients in both conservative and dialysis treatment.

## 6. α-Lipoic Acid

α-Lipoic acid (ALA) is a disulfide compound that acts as a coenzyme in pyruvate dehydrogenase and α-ketoglutarate dehydrogenase mitochondrial reactions [126]. Typical dietary sources of ALA are muscle meats, heart, kidney, and liver, and to a lesser degree, fruits and vegetables [127].

ALA and its reduced form, dihydrolipoic acid, have several antioxidant, anti-inflammatory, and metabolic activities.

ALA exerts direct antioxidant activity by chelating Cu^2+^, Zn^2+^, and Fe^2+^ [128,129], and it recycles other cellular antioxidants including CoQ10, vitamins C and E, and GSH. It also regulates the expression of numerous genes trough the inhibition of NFkB [130] and the induction of NRF2 [131].

As a modulator of peroxisome proliferator-activated receptor (PPAR)-α and -γ expression, ALA is implied in the regulation of glucose and lipid metabolism [131].

Moreover, it inhibits vascular calcification and vascular smooth muscle cell apoptosis by preserving mitochondrial functions and activating the PI3K/Akt pathway [132].

In a model of diabetic nephropathy, it reduced the progression of renal damage, glomerular mesangial matrix expansion, and glomerulosclerosis by restoring GSH, reducing MDA levels, and protecting mitochondrial function [133,134].

However, ALA supplementation in hemodialyzed patients showed uncertain results. A daily dose of 600 mg for eight weeks caused a reduction of CRP levels, without showing effects on MDA, total antioxidant status, total cholesterol, triglyceride, high-density lipoprotein cholesterol, or LDL levels [135]. Contrarily, in other studies, ALA combined with mixed tocopherols did not modify the level of several biomarkers of inflammation and oxidative stress [136,137,138,139].

Further studies, probably using mixed antioxidants and longer courses of treatment, could be useful to demonstrate the clinical utility of ALA supplementation in this patient population.

## 7. Selenium

Selenium is a non-metal element essential for human physiology, since it acts as a cofactor for several enzymes with an antioxidant role (glutathione peroxidase, thioredoxin reductase) [140]. It is obtained from meat, seafood, grains, cereals, fish (tuna and mackerel), and plants (garlic, onions, and broccoli), but the best source of selenium is Brazil nuts [141].

Several reports have demonstrated a selenium deficiency in CKD and dialyzed patients [142,143,144,145].

In patients with CKD, selenium supplementation increased the glutathione peroxidase activity [146], but unfortunately, this positive effect was detectable only if this agent was administered in the early stages of renal impairment [147].

Selenium supplementation (200 µg) for 12 weeks prevents the damage of DNA [148] and improved MDA levels [149], without showing any significant effects on inflammatory and thyroid functional biomarkers [150].

Moreover, 600 μg sodium selenide co-administered with 400 IU vitamin E before an HD session offsets the serum MDA increment induced by iron infusion [151].

At the moment, there is no indication to the use of selenium in CKD and additional, larger clinical studies are necessary to define its future potential employment in this clinical setting.

## 8. Green Tea

Green tea is composed of several polyphenolic compounds, but (−)-Epigallocatechin-3-gallate (EGCG) is the most abundant and effective as an antioxidant [152]. In fact, it is implicated in ROS scavenging, the inhibition of lipid peroxidation, and the chelation of metal ions such as copper (II) and iron (III) [153,154,155,156].

Recently these protective properties have become of interest specifically in kidney diseases, and EGCG has been tested in several animal models of glomerulonephritis [157]. Treatment with EGCG reduced proteinuria and serum creatinine, and it determined a marked improvement in several renal histological features. These effects seemed to be mediated by the direct and indirect effects of these agents on the redox cellular/biological system and the immune-inflammatory pathway [158,159,160]. In particular, EGCG increased renal NRF2 and glutathione peroxidase activity; reduced renal oxidative stress, NF-κB activation, and NLRP3 mRNA/protein expression as well as protein levels of mature caspase-1, IL-1β, and IL-18; and enhanced splenic regulatory T cell activity. EGCG-treated mice also showed a reduction in p-Akt, p-JNK, p-ERK1/2, and p-P38 as well as the restoration of peroxisome proliferator-activated receptor (PPAR)γ and sirtuin-1 (SIRT1) levels [158,159,160].

In streptozotocin-induced diabetic nephropathy, EGCG administration for 50 days improved renal function as well as reduced renal AGE accumulation, lipid peroxidation, and fibronectin levels [161].

In animal models of unilateral urethral obstruction, EGCG caused the upregulation and nuclear translocation of NRF2, with the consequent enhancement of antioxidant enzymes such as γ-glutamylcysteine synthetase [162].

EGCG significantly reduced uremic toxins, such as methylguanidine levels, in a dose-dependent manner in rats with adenine-induced renal failure [163].

In an ischemia reperfusion injury model, EGCG treatment, via monocyte chemoattractant protein-1 (MCP-1) and transforming growth factor (TGF-β) downregulation and heme oxygenase 1 (HO-1) augmentation, protected the kidneys from a massive infiltration of macrophages [164] and the development of chronic renal alterations.

It must be noted, however, that all of the abovementioned studies are speculative and no large clinical trials have been published in nephrology.

## 9. Resveratrol

Resveratrol (3,5,4′-trihydroxystilbene) is a naturally occurring polyphenolic compound present in more than 70 species of plants, with the greatest amount found in grapes, berries, red wine, and peanuts [165].

Resveratrol can directly scavenge ROS and modulate the expression and activity of antioxidant enzymes such as SOD, glutathione peroxidase, and catalase, through transcriptional regulation via NRF-2, activator protein 1 (AP-1), forkhead box O (FOXO), and SP-1 [166,167]. Moreover, it is an activator of SIRT1, an NAD^+^-dependent deacetylase of histones, which results in reduced transcriptional activity. SIRT1 plays a key role in responding to nutritional and environmental perturbations such as fasting, calorie restriction, starvation, and nutrient deprivation, as well as in oxidative stress conditions [168,169]. SIRT1 activates peroxisome proliferator-activated receptor gamma coactivator 1-α (PGC-1α) and attenuates sterol-regulatory-element-binding protein (SREBP) activity, resulting in increased fatty acid oxidation, reduced lipogenesis and cholesterolgenesis, and improved glucose homeostasis and mitochondrial function [170,171]. Moreover, it deacetylates FOXO, p53, hypoxia-inducible factor (HIF)-1α, and NFkB, mediating antiapoptotic, antioxidative, and anti-inflammatory effects [172,173].

Due to its cytoprotective effects, resveratrol has been largely tested in animal models of chronic renal diseases. In streptozotocin-induced diabetic rats, resveratrol (5 or 10 mg/kg administered orally) improved renal dysfunction and oxidative stress, and attenuated cytokine levels [174,175].

Resveratrol, then, seemed to prevent in vitro high glucose-induced mesangial cell proliferation and fibronectin expression by inhibiting JNK and NF-κB, as well as NADPH oxidase activity and ROS production [176]. These results were in line with more recent studies that demonstrated a positive effect of this agent in podocytes of db/db mice through the activation of the autophagic pathway [177].

Resveratrol, through its antioxidant mechanisms and the deacetylation of Smad3, directly prevents EMT and renal fibrosis [176,178,179,180,181], and inhibits CKD-induced skeletal muscle atrophy mediated by NF-κB [182].

Moreover, by enhancing the AMPK/SIRT-1/PGC-1α axis [183] this agent could attenuate mitochondrial dysfunction and aldosterone-induced podocyte injury [184].

In other animal models of AKI (cisplatin nephropathy, ischemia-reperfusion, and sepsis-related acute renal damages), resveratrol demonstrated a considerable renal protective effect by normalizing Nrf2 renal expression, enhancing antioxidant factors expression (HO-1, GST), and reducing inflammatory mediators (TNF-α, IL-6) [185,186,187].

Unfortunately, although resveratrol has demonstrated low adverse effects, no study has been developed to evaluate its efficacy in CKD patients, probably because its low bioavailability [188]. In the future, the employment of this agent in combination with other compounds could probably permit its use to counteract the progression to end-stage renal disease.

## 10. Curcumin

Curcumin is the active element of curcuma longa (or turmeric), a perennial herbaceous plant member of the ginger family (Zingiberaceae), prevalent in India, China, and Southeast Asia [189].

In Ayurvedic and Chinese medicine, curcumin is used as anti-inflammatory, antioxidant, antibacterial, and antimicrobial reagent, as well as to treat chronic diseases [190,191,192,193,194].

Its antioxidant properties are due to both a direct scavenger activity and the upregulation of antioxidant and cytoprotective genes. Curcumin is able to directly scavenge superoxide anions, hydroxyl radicals, H_2_O_2_, singlet oxygen, nitric oxide, and peroxynitrite [195,196,197,198], probably by means of phenolic groups in its molecular structure. Curcumin also has an indirect antioxidant ability mediated by the induction of the expression of cytoprotective enzymes such as SOD, catalase [199], glutathione reductase, HO-1 [200], glutathione *S*-transferase, NAD(P)H:quinone oxidoreductase 1 [201], and γ-glutamylcysteine ligase [202].

Several studies have reported the reno-protective effects of curcumin in a mouse model of diabetic nephropathy. Administration of curcumin (15–150 mg/kg/day for two to eight weeks) ameliorated renal function through the upregulation of antioxidant enzymes, inhibition of NADPH oxidase and NFkB, and it reduced macrophages infiltration and proinflammatory cytokines together with antifibrotic activities [203,204,205,206,207]. 

Likewise, in an animal model of CKD, curcumin significantly reversed proteinuria, hypertension, interstitial fibrosis, fibrotic glomeruli, tubular atrophy, and mesangial expansion [208,209,210,211,212,213]. Interestingly, the preservation of mitochondrial dynamics, bioenergetics, and oxidative stress has been recently demonstrated in a rat model of CKD, which may be associated with ameliorated renal function [214].

Moreover, curcumin improved structural and functional manifestations of cardiac injury associated with renal failure, in part through the inhibition of NLRP3 inflammasome and the preservation of mitochondrial function [215,216,217].

Khajehdehi et al. showed that an oral supplementation of 500 mg turmeric (of which 22.1 mg was the active ingredient curcumin) for three months has strong renal protective effects in lupus nephritis and type 2 diabetic nephropathies [218,219].

However, although it is a promising agent for the treatment of chronic glomerulopathies and tubular renal dysfunctions, at the moment, the clinical evidences are not adequate to justify a large utilization of this compound in our nephrology patients.

## 11. Omega-3 Polyunsaturated Fatty Acids

Omega-3 polyunsaturated fatty acids (omega-3 PUFAs) are a class of essential long-chain fatty acids obtained primarily from dietary sources.

The two most bioactive and extensively studied omega-3 fatty acids are eicosapentaenoic acid (EPA) and docosahexaenoic acid (DHA) [220].

Omega-3 PUFAs exert anti-inflammatory activity by modifying the expression of adhesion molecules, chemotactic factors, and proinflammatory cytokines (TNF-α, IL-1β, and IL-6) [221,222,223,224].

Another mechanism is the prevention of the conversion of arachidonic acid into proinflammatory eicosanoids such as prostaglandin (PG) and leukotriene (LT) [225], and serving as an alternative substrate to produce less potent 5-series LTs, 3-series PGs, and thromboxanes [226]. In addition, omega-3 PUFA-derived resolvins such as resolvin E1 (RvE1) and D-series resolvins and protectin D1 from DHA have potent anti-inflammatory actions [227,228].

These compounds maintain the structure and function of cell and organelle membranes [229], participating in membrane fluidity, ion channels transport (sodium, potassium, and calcium) [230], and mitochondrial biogenesis [231].

In addition to anti-inflammatory properties, omega-3 fatty acids also have antioxidant effects.

They enhance endogenous antioxidant defense systems such as GSH through the increased activity of γ-glutamyl-cysteinyl ligase, glutathione reductase, and glutathione *S*-transferase [232], and compete with arachidonic acid at COX2 and xanthine oxidase sites, reducing ROS synthesis [233].

In animal models of CKD, EPA and DHA supplementation reduce inflammation, fibrosis, and oxidative stress [234,235,236].

In CKD and dialysis patients, the administration of omega-3 PUFAs can reduce inflammation associated with CKD progression through the upregulation of E- and D-series resolvins [237], together with the reduced level of endothelial chemokines, RANTES, and MCP-1 [238].

At the same time, recent studies suggest a benefit of omega-3 PUFAs supplementation in ameliorating uremic symptoms (particularly pruritus) [239] and reducing hypertension [240,241].

In a randomized controlled clinical trial on 78 patients affected by IgA nephropathy, PUFAs associated with renin-angiotensin system blockers were more effective than renin-angiotensin system blockers alone in reducing proteinuria. Unfortunately, the small number of patients, the short time of observation (six months) and the single-center nature of the study limits the power of these conclusions [242].

Because of the few strong evidences of its benefit in CKD, additional studies are warranted to assess the real efficacy of these agents in slowing the progression of renal failure and to establish the formal intake recommendations and dosing in the CKD patient population (particularly in dialysis).

## 12. Conclusions

CKD is associated with enhanced oxidative stress that is a well-known risk factor for the onset/development of severe systemic complications and cardiovascular diseases. Several studies report a higher level of oxidative stress markers together with reduced antioxidants in pre-dialysis patients. This condition is exacerbated during the progression of renal failure and in renal replacement therapy. Several biological mechanisms contribute to oxidative stress, including mitochondrial activity, xanthine oxidase, and NADPH oxidase. Thus, in recent years the administration of antioxidants, both food-derived and through drugs with additional antioxidant effects, have demonstrated positive effects. Many of these compounds have direct ROS scavenger properties due to their molecular structure (l-carnitine, vitamin E, vitamin C, α-lipoic acid, green tea, resveratrol, curcumin), others also have indirect antioxidant effects mediated by the upregulation of antioxidant enzymes (l-carnitine, green tea, α-lipoic acid, resveratrol, curcumin) or by additional anti-inflammatory properties (vitamin E, resveratrol, curcumin, omega-3).

However, the great limit of most of these studies is the low sample size and short-term follow-up. As a consequence, none of these molecules have been introduced into usual clinical practice. Therefore, future prospective and comparative studies analyzing the co-administration of different antioxidants with long-term follow-up are warranted.
